# Crizotinib-induced antitumour activity in human alveolar rhabdomyosarcoma cells is not solely dependent on ALK and MET inhibition

**DOI:** 10.1186/s13046-015-0228-4

**Published:** 2015-10-06

**Authors:** Francesca Megiorni, Heather P. McDowell, Simona Camero, Olga Mannarino, Simona Ceccarelli, Milena Paiano, Paul D. Losty, Barry Pizer, Rajeev Shukla, Antonio Pizzuti, Anna Clerico, Carlo Dominici

**Affiliations:** Department of Paediatrics and Infantile Neuropsychiatry, Sapienza University, Viale Regina Elena 324, 00161 Rome, Italy; Department of Oncology, Alder Hey Children’s NHS Foundation Trust, Eaton Road, L12 2AP Liverpool, UK; Department of Experimental Medicine, Sapienza University of Rome, Viale Regina Elena 324, 00161 Rome, Italy; Department of Paediatric Surgery, Alder Hey Children’s NHS Foundation Trust; Academic Paediatric Surgery Unit, University of Liverpool, Eaton Road, L12 2AP Liverpool, UK; Department of Perinatal and Paediatric Pathology, Alder Hey Children’s NHS Foundation Trust, Eaton Road, L12 2AP Liverpool, UK

**Keywords:** Crizotinib, ALK, MET, Alveolar rhabdomyosarcoma, G2/M arrest, IGF1R

## Abstract

**Background:**

Rhabdomyosarcoma (RMS) is the most commonly diagnosed malignant soft tissue tumour in children and adolescents. Aberrant expression of Anaplastic Lymphoma Kinase (ALK) and MET gene has been implicated in the malignant progression of RMS, especially in the alveolar subtype. This observation suggests that crizotinib (PF-02341066), a kinase inhibitor against ALK and MET, may have a therapeutic role in RMS, although its antitumour activity in this malignancy has not yet been studied.

**Methods:**

RH4 and RH30 alveolar RMS (ARMS) cell lines were treated with crizotinib and then assessed by using proliferation, viability, migration and colony formation assays. Multiple approaches, including flow cytometry, immunofluorescence, western blotting and siRNA-based knock-down, were used in order to investigate possible molecular mechanisms linked to crizotinib activity.

**Results:**

*In vitro* treatment with crizotinib inhibited ALK and MET proteins, as well as Insulin-like Growth Factor 1 Receptor (IGF1R), with a concomitant robust dephosphorylation of AKT and ERK, two downstream kinases involved in RMS cell proliferation and survival. Exposure to crizotinib impaired cell growth, and accumulation at G2/M phase was attributed to an altered expression and activation of checkpoint regulators, such as Cyclin B1 and Cdc2. Crizotinib was able to induce apoptosis and autophagy in a dose-dependent manner, as shown by caspase-3 activation/PARP proteolytic cleavage down-regulation and by LC3 activation/p62 down-regulation, respectively. The accumulation of reactive oxygen species (ROS) seemed to contribute to crizotinib effects in RH4 and RH30 cells. Moreover, crizotinib-treated RH4 and RH30 cells exhibited a decreased migratory/invasive capacity and clonogenic potential.

**Conclusions:**

These results provide a further insight into the molecular mechanisms affected by crizotinib in ARMS cells inferring that it could be a useful therapeutic tool in ARMS cancer treatment.

**Electronic supplementary material:**

The online version of this article (doi:10.1186/s13046-015-0228-4) contains supplementary material, which is available to authorized users.

## Background

Rhabdomyosarcoma (RMS) is the most common malignant soft tissue tumour in childhood, representing approximately 50 % of all sarcomas and 4–5 % of malignant solid tumours in children aged 0–14 years [1, 2]. The two major histological subtypes, alveolar rhabdomyosarcoma (ARMS) and embryonal rhabdomyosarcoma (ERMS), differ in prevalence, body location, clinical features and outcome [3]. ARMS generally carries either t(2;13)(q35,q14) or t(1;13)(p36;q14) translocations, which generate PAX3/FOXO1 and PAX7/FOXO1 fusion proteins, respectively [4, 5], whilst a wide range of genetic aberrations, including loss of heterozygosity at 11p15.5 [6], have been identified in ERMS. Even gene expression profiles widely differ in ARMS and ERMS tumours [7]. Recent studies have demonstrated aberrant ALK (anaplastic lymphoma kinase) expression especially in PAX3/7-FOXO1-positive ARMS samples [8–11]. ALK is a member of the insulin receptor family of receptor tyrosine kinases (RTKs) and is up-regulated during embryonic development of the nervous system, being maintained at low levels in adult tissues [12]. MET, another RTK normally activated in development and tissue regeneration [13], was found to be highly expressed in RMS cell lines [14]. Aberrant expression, amplification, translocations or mutations involving ALK and MET genes have an important role in driving tumourigenesis through the activation of multiple pathways, including the PI3K/AKT signalling cascade, which controls many cellular functions including survival, proliferation, differentiation, adhesion and migration [11–18]. Several studies have reported that ALK or MET over-expression and constitutive kinase activation correlate with advanced stage and may be predictive of poor clinical outcome in several neoplasia, such as neuroblastoma, gastrointestinal carcinoma and non-small-cell lung cancer [19–23]. Crizotinib (PF-02341066) is a small molecule initially identified as a highly potent inhibitor of ALK and MET receptor tyrosine kinases [24, 25]. Subsequent studies have shown that crizotinib is also able to block the activation of other RTKs involved in tumour development/progression, such as ROS1 and PDGFR-alpha [26, 27]. The antitumour activity of crizotinib in cancer clinical trials has been significant [25, 28], leading to the Food and Drug Administration (FDA) approval as an integral addition to clinical treatment of patients with non-small cell lung cancer (NSCLC) carrying ALK translocations [29, 30]. More recently, crizotinib has also been proposed as a promising drug in soft tissue sarcomas [28], but the possible activity in RMS has not yet been elucidated.

Since ALK/MET-positive RMSs may be sensitive to ALK/MET tyrosine kinase inhibitors, the present study investigated whether crizotinib was able to abolish the oncogene-dependent signalling by affecting proliferation and survival in this subset of RMS. RH4 and RH30, two human ARMS cell lines harbouring PAX3-FOXO1 translocation, and with raised ALK and MET expression [11, 14], were treated with crizotinib to evaluate the effects on cell proliferation, apoptosis, autophagy, colony formation ability and cell cycle as well as on several specific molecular pathways. Incubation with crizotinib resulted in a dose-dependent reduction in tumour cell growth, with a clear arrest in G2/M phase and - using high concentrations (i.e. 5 μM) - a pronounced apoptosis and autophagy. Crizotinib blocked the activation of ALK, MET and IGF1R, with a concomitant inhibition of the downstream mediator AKT, whose constitutive phosphorylation is associated with malignant transformation and poor outcome in RMS [31]. This study is the first to describe the *in vitro* activity of crizotinib in RMS tumours, this suggesting that this molecule may be a potential therapeutic agent that effectively controls ARMS growth by inhibiting ALK, MET and IGF1R pathways.

## Methods

### Compound

Crizotinib, also known as PF-02341066, was supplied as lyophilized powder by Cell Signalling Technology (Danvers, MA) and reconstituted in dimethyl sulfoxide (DMSO, Sigma, St. Louis, MO) to a final concentration of 2 mM. Aliquots were conserved at −20 °C.

Human recombinant IGF1 was purchased as lyophilized powders (PeproTech EC Ltd, UK) and reconstituted in sterile deionised H_2_O and stored in aliquots at −20 °C.

### Cell cultures

Human ARMS (RH4 and RH30) and ERMS (RD and RD18) cells [32, 33] were cultured in complete medium, i.e. DMEM-HG (Carlsbad, CA), supplemented with 10 % Foetal Bovine Serum (FBS) (Gibco), 2 mM L-glutamine (Gibco), 100 IU/mL penicillin and 100 μg/ml streptomycin (Gibco). All cell lines were maintained at 37 °C in 5 % CO_2_.

### Tumour samples

Six RMS tumour samples, 3 ARMSs and 3 ERMSs, were obtained at diagnosis before any treatment from children admitted to the Department of Oncology at Alder Hey Children’s NHS Trust, Liverpool. Histopathological diagnosis was confirmed using immunohistochemistry. ARMS were investigated for PAX3/7-FOXO1 translocations using standard FISH analysis, and all were positive. Institutional written informed consent was obtained from the patient’s parents or legal guardians. The study underwent ethical review and approval according to the local institutional guidelines (Alder Hey Children’s NHS Foundation Trust Ethics Committee, approval number 09/H1002/88).

### RNA extraction and RT-PCR

Total RNA was isolated from the four cell lines using TRIzol reagent (Invitrogen, Carlsbad, CA) according to the manufacturer’s instructions. One microgram of total RNA was reverse transcribed using the High Capacity cDNA Reverse Transcription Kit (Life Technologies, Carlsbad, CA, USA). PCR was conducted as previously described [9]. Subsequent PCR reactions were performed with gene-specific primers, designed from the human ALK (ALK forward 5′-GCTGAGCAAGCTCCGCACCTCGAC-3′ and ALK reverse 5′-CCCGCCATGAGCTCCAGCAGGATG-3′) and MET (MET forward 5′-GAGCGCTTTGTGAGCAGATG-3′ and MET reverse 5′-AACCAGTGGAGAAGTCAGCG- 3′) exonic sequences. GAPDH housekeeping gene was used as control. RT-PCR products were resolved on a 1.5 % agarose gel.

### Cell proliferation assays

RH4 and RH30 cell proliferation was measured using the 3-[4,5-dimethylthiazol-2-yl]-2,5 diphenyl tetrazolium bromide (MTT) assay. RH4 and RH30 cells (5×10^3^) were seeded in sexuplicates into 96-well plates 24 h before treatment with crizotinib at concentrations ranging from 0.01 to 5 μM. Control cells were treated with DMSO at the maximum amount used to deliver crizotinib. Treatment medium was replaced every day with a medium containing a fresh drug dilution. At 72 h of crizotinib exposure, 0.5 mg/ml MTT solution was added to each well for 3 h. After incubation, 200 μl of DMSO were added to each well and mixed thoroughly. Absorbance was measured at 540 nm, with a reference wavelength of 630 nm, using a plate reader and the readings were plotted as a mean of OD_treatment_/OD_control_ ± standard deviation (SD).

For cell number count, RH4 and RH30 cells were seeded onto 12-well plates and treated with 1.5 μM crizotinib or 0.075 % DMSO for 72 h. Direct counting of living cells was performed by labelling cells with trypan blue (1:1) exclusion dye (Sigma). Trypan blue-negative cells were regarded as viable cells.

### Cell cycle analysis

RH4 and RH30 cells were incubated in 6-well cell culture plates overnight to allow cell adhesion. After incubation, cells were treated for 24, 48 or 72 h with 1.5 μM or 5 μM crizotinib. At the end of each treatment, cells were trypsinised, washed with PBS, fixed in 75 % ice-cold ethanol and stored overnight at 4 °C. Cells were washed with PBS, treated with 100 μg/mL RNAse A for 30 min at 37 °C and then stained with 50 μg/mL Propidium Iodide (PI) solution. Samples subjected to flow cytometric analysis were loaded into a BD FACSCalibur (BD Biosciences, Franklin Lakes, NJ) and PI fluorescence was detected in FL2 with active Double Discrimination Module using CellQuest Pro (BD Biosciences). DNA cell cycle analysis of flow cytometric data was carried out using the software ModFit LT 3.0 (Verity Software House). Three independent experiments were performed for each treatment.

### Morphological assessment of crizotinib-treated cells

An inverted phase contrast microscope was used to observe the morphological changes of the cells treated or untreated with 1.5 μM crizotinib. After being cultured for 48 h, cells were stained by the standard Giemsa method. Briefly, cells were fixed by cold methanol for 5 min and stained in 10 % Giemsa solution (Sigma) for 15 min at RT. After rinsing in tap water, cells were air-dried and photographed under a light microscope at 40× magnification. The nuclei were stained blue/violet.

### Apoptosis analysis

Apoptosis was analysed by flow cytometry using PE Annexin V Apoptosis Detection Kit I (BD Biosciences). Briefly, RH4 and RH30 cells were seeded overnight in 6-well plate and treated with crizotinib for 48 h at 1.5 μM or 5 μM. Control cells were treated with DMSO at the maximum amount used to deliver crizotinib. DMSO had no toxic effects on RH4 and RH30 cell lines in total cell count experiments with culture media only, DMSO only and crizotinib (data not shown). Floating and attached cells were collected, washed twice in cold 1× Annexin V Binding Buffer and suspended in 1× Annexin V Binding Buffer at 10^6^ cells/ml. Approximately 10^5^ cells were stained with Annexin V and 7-Amino-Actinomycin (7-AAD) for 15 min at RT in the dark according to the manufacturer’s instructions. Annexin V and 7-AAD fluorescence intensities of control or treated samples were analysed using a BD FACSCalibur (BD Biosciences). Data were collected analysed using Cell Quest Pro software (BD Biosciences).

Caspases-3/7 activities were measured using Caspase-glo 3/7 kit assay (Promega, Madison, WI, USA). Briefly, RH4 and RH30 cells were seeded overnight in a 96-well plate and treated with crizotinib at 0–1.5–5 μM. After 48 h, 100 μl of Caspase-glo 3/7 reagent were added to each well and cells were incubated in the dark for 1 h at RT. Luminescence was read in a Glomax luminometer (Promega) and results were expressed as fold change of caspase 3/7 activity to respective control cells.

### Migration and invasion assays

RH4 and RH30 cells (5×10^4^ in 24-well plates) were treated in complete medium with 1.5 μM crizotinib or with DMSO, as a control, for 24 h before plating into BD FalconTM Cell Culture Inserts (Corning, Tewksbury, MA). Chambers with cells contained medium without serum, whilst the lower well had DMEM supplemented with 10 % FBS, used as chemo-attractant. After 24 h, cells which had not migrated were removed by swabbing, whilst the migrated cells at the base of the inserts were fixed in 100 % methanol and stained with 0.1 % crystal violet dye.

For tumour invasion assay, RH4 and RH30 cells (5×10^4^ in 24-well plates) treated with or without 1.5 μM crizotinib for 24 h were plated into Matrigel-coated chambers (BD Biosciences). Medium in the upper chamber was supplemented without FBS, while FBS concentration was 10 % in the lower chamber. After 24 h, the non-invasive cells on the upper surface of the membrane were removed with a cotton swab, whilst cells able to invade through the Matrigel matrix and attached to the lower surface of the chamber were fixed and stained with crystal violet.

Cells were photographed under a light microscope at 20× magnifications; 8 randomly selected fields were examined and counted. The average number of migrated cells was calculated.

### Soft agar assay

For anchorage-independent colony formation assays, 2 ml of 0.7 % low-melting agarose in DMEM-HG with 10 % FBS were poured into 6-well plates. Subsequently, 2 ml of top agar consisting of 0.35 % low-melting agarose in complete medium, 3×10^4^ cells and 1.5 μM crizotinib or DMSO were added to each well. After solidification, each well was covered with 1 ml of culture medium (in the presence or absence of crizotinib), which was refreshed every 3 days. Cells were allowed to grow at 37 °C and 5 % CO_2_. After 21 days, colonies were stained with 0.05 % crystal violet for 1 h and photographed under an inverted light microscope. Colonies which exceed an arbitrary size ≥ 50 μm in diameter were counted in drug-treated and mocked-control cultures.

### Colony formation assay

For anchorage-dependent colony formation assays, RH4 and RH30 were plated in 6-well plates at 5×10^3^ cells/well. After 24 h, 1.5 μM crizotinib or DMSO were added to the wells. Cells were cultured in the presence or absence of crizotinib for 8 days and then colonies were visualized by light microscopy. Colonies which exceed an arbitrary size ≥ 50 μm in diameter were counted in drug-treated cultures relative to mocked-control cultures.

### Immunoprecipitation and western blotting

RH4 and RH30 cells were plated in 6-well plates (4×10^5^ cells/well) and grown overnight to allow cell adhesion. Cells were treated for 24 h with 1.5 μM crizotinib and then lysed in RIPA buffer (50 mM Tris–HCl pH 7.4, 150 mM NaCl, 1 mM EDTA, 1 % NP-40, 1 mM PMSF, 50 mM NaF, 1 mM Na_3_VO_4_, and 1× protease inhibitors) for 20 min. Samples were cleared at 14,000 rpm for 10 min and quantified using the Biorad Protein Assay Kit (Biorad, Berkeley, CA). For immuniprecipitation, total proteins (800 μg) were extracted with IP-buffer (25 mM Hepes, 400 mM NaCl, 1.5 mM MgCl_2_, 2 mM EDTA, 0.5 % Triton-X, 3 mM DTT, 1 mM Na_3_VO_4_, 1 mM PMSF, 1× protease inhibitors) and incubated with primary antibody (2.5 μg) overnight at +4 °C. A/G-conjugated agarose beads (Santa Cruz Biotechnology, Dallas, TX) were added and incubated for 2 h at 4 °C. Immunocomplexes were washed 3 times with IP-buffer. For Western blotting, total protein extracts (30–100 μg) were resolved on 8–15 % SDS-PAGE gels and transferred onto PVDF membranes (Merck Millipore, Guyancourt, F). Blots were blocked in 5 % not-fat milk or BSA and incubated over-night at +4 °C with the following primary antibodies: phospho (p)-ALK (1:500), p-MET (1:500), p-ERK (1:3000), p-AKT (1:2000), p-IGF1R (1:1000), ALK-XP (1:2000), AKT (1:2000), ERK (1:2000), IGF1R (1:1000), cleaved caspase-3 (1:200), cleaved PARP (1:1000) by Cell Signalling Technology; MET (1:1000), Cyclin B1 (1:1000), Cyclin D3 (1:250), p-Cdc2 (1:300), Cdc25A (1:1000), Cdc25C (1:1000), p27 (1:500) and Cdk4 (1:1000) by Santa Cruz Biotechnology; p62/sequestosome 1 (1:1000, BD Biosciences); LC3 (1:1000, Sigma). Antibody against tubulin (1:20000, Sigma) was used as a loading control. Membranes were then incubated in appropriate horseradish peroxidase-conjugated secondary antibodies (1:4000 dilution, Santa Cruz Biotechnology). Protein signals were detected using WesternBright ECL kit (Advansta, Menlo Park, CA), according to the manufacturer’s instructions, and visualized by ChemiDoc XRS^+^ (Bio-Rad, Texas, US).

### Immunofluorescence

RH4 and RH30 cells (10^4^), plated on coverslips into 24-well plates, were allowed to attach overnight and then incubated for 24 h in the presence of 1.5 μM crizotinib or DMSO, as control, at 37 °C. Cells were fixed in 4 % paraformaldehyde (PFA) in PBS for 30 min at RT, followed by treatment with 0.1 M glycine in PBS for 20 min at RT and with 0.1 % Triton X-100 in PBS for additional 5 min at RT to allow permeabilization. Cells were incubated with primary antibodies against p21, Cdc25C, Cyclin B1, Cdc2 or p-Cdc2 (1:20 dilution in PBS; Santa Cruz Biotechnology) for 1 h at RT. After washing in PBS, cells were incubated with appropriate FITC-conjugated or Texas Red-conjugated secondary antibodies (1:200 dilution in PBS; Jackson Immunoresearch, West Grove, PA) for 30 min at RT in the dark. Nuclei were stained with using 4′, 6-diamido-2-phenylindole dihydrochloride (DAPI) (1:10000 in PBS; Sigma). TRITC-phalloidin (Sigma), identifying filamentous actin of the cellular cytoskeleton, was used at 1:50 dilution in PBS. The single-stained and merged images were acquired with Zeiss ApoTome and Axiovision software (Carl Zeiss, Jena, Germany) using a 40× magnification.

To monitor microtubule dynamics, cells were treated with 1.5 μM crizotinib or 50 ng/ml nocodazole, as positive control. Cells were fixed after 4 h, stained with anti-alpha-tubulin (1:2000 in PBS; Sigma)/FITC-anti-mouse antibody.

For assessment of the Annexin V-FITC/PI staining in apoptotic cells, RH4 and RH30 cells, plated on coverslips and treated with crizotinib (1.5 and 5 μM) for 48 h, were washed with 1× binding buffer and incubated with Annexin V-FITC and PI for 15 min at RT in the dark. Cells were fixed in 4 % PFA in 1× binding buffer for 30 min at RT, followed by 1× binding buffer washing for three times. Control cells were treated with DMSO at the maximum amount used to deliver crizotinib.

All the single-stained and merged images were acquired with Zeiss ApoTome and Axiovision software (Carl Zeiss, Jena, Germany) using a 40× magnification.

### Detection of reactive oxygen species (ROS)

The accumulation of ROS in RH4 and RH30 cells was determined using the general oxidative stress indicator H_2_DCFDA that fluoresces upon oxidation. Cells, treated with crizotinib (1.5 and 5 μM) for 24 h, were incubated at 37 °C in medium containing 5 μM H_2_DCFDA (Sigma). Control cells were treated with DMSO at the maximum amount used to deliver crizotinib. After 40 min, cells were washed with PBS and fluorescent intensity was detected by FACSCalibur in FL-1 channel. In some experiments, cells were pre-incubated with or without NAC (5 mM) for 1 h, and then treated with or without crizotinb for 24 h, followed by loading with 5 μM of H_2_DCFDA.

### siRNA transfection

ALK and MET siRNA (Ambion) were transfected into RH4 and RH30 cells with RNAiMax (Invitrogen) according to the manufacturer’s instructions. Negative Control (NC) siRNA (Ambion), having no significant sequence similarity to mouse, rat, or human gene sequences, was used as in mocked-control cells. Forty-eight hours after transfection, cells were submitted to different molecular techniques for further analysis.

### Statistical analysis

Statistical significance was assessed by Student’s t-tests and probability (p) values of less than 0.05 were accepted as significant. All the experiments were done in triplicates and repeated three times unless mentioned otherwise.

## Results

### ALK and MET are differentially expressed in human ARMS *vs.* ERMS cell lines and tumour samples

**Fig. 1:**
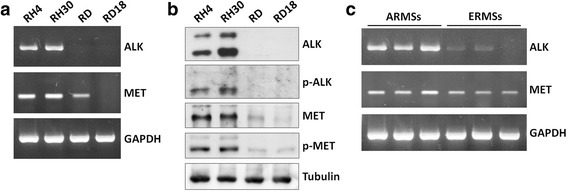
ALK and MET expression in RMS cell lines and tumour samples. **a** RT-PCR analysis showing the expression of ALK and MET mRNAs in RH4 and RH30, two ARMS cell lines, but faintly in RD and RD18, two ERMS cell lines. GAPDH expression used as the internal control. **b** Western blotting experiments showing higher levels of both total ALK and MET proteins in ARMS than in ERMS cells. The activated forms of both ALK and MET proteins were observed in ARMS samples using the specific p-ALK (Tyr1604) and p-MET (Tyr1234/Tyr1235) antibodies, respectively. Tubulin expression was used as the internal control. **c** Expression of ALK and MET mRNAs measured by RT-PCR in a panel of ARMS and ERMS tumour samples

The ALK and MET expression at mRNA and protein levels in two ARMS (RH4 and RH30) and two ERMS (RD and RD18) cell lines was evaluated. RT-PCR experiments showed that ARMS samples had a marked expression of ALK and MET transcripts in comparison to ERMS cells (Fig. 1a). Western blotting analysis showed higher levels of both total ALK and MET proteins in RH4 and RH30 cells than in RD and RD18 cells (Fig. 1b). ALK protein was detected as both an approximately 200 kDa band, reflecting the full-length form, and a second band of about 140 kDa (Fig. 1b), which may represents a splice variant or a degraded product of ALK [33]. Similarly, activation of ALK and MET proteins (p-ALK and p-MET) was evident only in ARMS cells (Fig. 1b) even if basal phosphorylation of ALK receptor was weakly detectable in accordance with its recently demonstrated susceptibility to intracellular phosphatases [11]. RT-PCR analysis performed in a panel of 6 RMS tumours (3 ARMSs and 3 ERMSs) confirmed that ALK and MET mRNAs are expressed at significantly higher levels in ARMS compared to ERMS (Fig. 1c). Taken together, these data suggest that ALK and MET expression is up-regulated in ARMS.

### Crizotinib inhibits cell proliferation and viability in ARMS cell lines

**Fig. 2:**
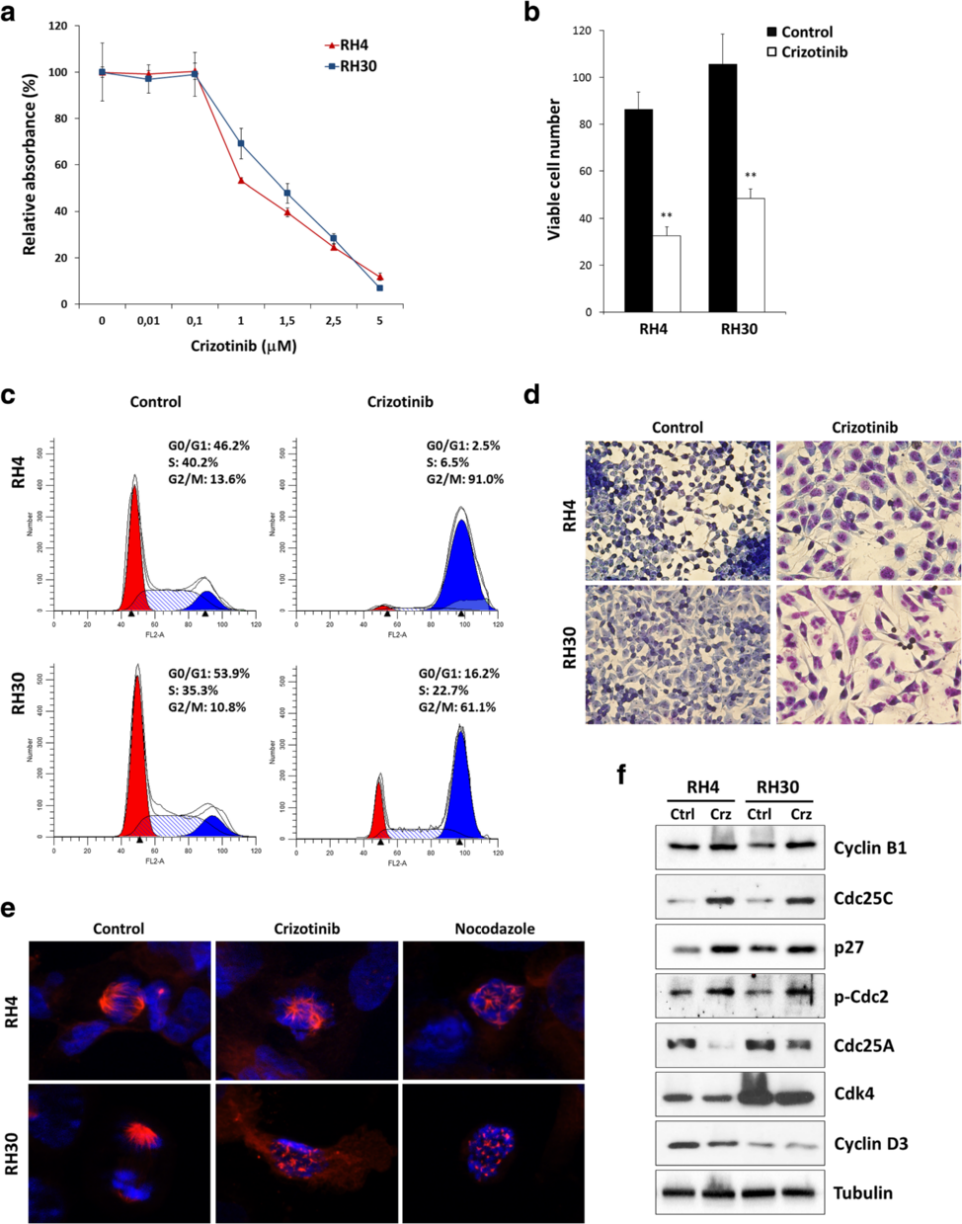
Effects of crizotinib on cell proliferation and morphology of RH4 and RH30 cell lines. **a** RH4 and RH30 cells were incubated for 72 h with crizotinib at the indicated concentrations. The MTT assay was performed to assess relative cell numbers, which were plotted as a percentage of cell viability calculated with respect to control cells treated with DMSO alone (0 μM considered at 100 %). Each point is the mean of nine replicate wells ± standard deviation (SD) and is representative of three independent experiments. **b** RH4 and RH30 were plated on 12-well plates and treated with 1.5 μM crizotinib for 72 h. Control represents DMSO-treated cells. Viable cells were counted by trypan blue exclusion staining and each bar represents the mean value of three independent experiments ± SD. Statistical significance: **, *p* < 0.01. **c** Flow cytometry data represented as histograms showing percentages of cells in G0/G1, S and G2/M phases in RH4 and RH30 cells after treatment with 1.5 μM crizotinib for 48 h. Control cells received DMSO at the same concentration. Data are average values of three independent experiments. **d** Giemsa staining showing morphologic changes observed in RH4 and RH30 after 48 h of treatment with 1.5 μM crizotinib or mocked control. Cellular morphology was analysed under light microscope at 40× magnification. **e** Immunofluorescence analyses showing the effect of crizotinib on microtubule dynamics. Normal bipolar spindle formation was altered in RH4 and RH30 cells treated for 24 h with crizotinib. Cells were stained for tubulin (red) and DNA (blue). Nocodazole was used as positive control for microtubule depolymerisation. Control cells were treated with DMSO. **f** Western blot analyses of a panel of cell cycle regulatory proteins in RH4 and RH30 cells treated with 1.5 μM crizotinib (Crz). Control cells (Ctrl) were treated with DMSO. Tubulin expression was used as the internal control

Since significant ALK and MET expression was only detected in ARMS samples, the effect of crizotinib, an ATP competitive, MET and ALK inhibitor, was evaluated on the growth of RH4 and RH30 ARMS cell lines. Cells were exposed for 72 h to increasing concentrations of the drug, with values ranging from 0 to 5 μM. Cell growth and viability were clearly inhibited in a dose-dependent manner, with IC_50_ values of about 1.5 μM (Fig. 2a), which are within the range of clinical relevance [25]. Direct counting for living cells using the trypan blue dye exclusion test confirmed that crizotinib induced a significant reduction in cell number (Fig. 2b).

### Crizotinib inhibits cell growth and modulates G1/S and G2/M cell cycle signalling pathways in ARMS cell lines

To further determine whether the crizotinib-dependent decrease in ARMS cell growth was due to alterations in cell cycle progression, flow cytometry analysis was performed in RH4 and RH30 cells treated with or without crizotinib. Based on propidium iodide staining of cellular DNA content, almost all cells arrested in G2/M phase (4n) when treated with 1.5 μM crizotinib with a corresponding decrease of cell percentage in both G1 (2n) and S phases, while untreated cells rapidly divided and progressed through the cell cycle at high rates (Fig. 2c). Indeed, a high proportion of ARMS cells accumulated in G2/M after 24 h, with a maximum 4n-peak at 48 h and the appearance of extra populations of hyperdiploid cells (>4n) after 72 h of crizotinib-exposure (data not shown). c (0.01, 0.5 and 1 μM) did not significantly affect the cell cycle whilst higher crizotinib concentrations (2.5 and 5 μM) profoundly increased the number of G2/M phase cells and sub-G1 (apoptotic) population (data not shown), confirming a dose-dependent G2/M cell growth arrest in both RH4 and RH30 cells.

Crizotinib exposure clearly affected the morphologic appearance of both RH4 and RH30 cells, as confirmed by Giemsa staining (Fig. 2d), with cells becoming larger, less dense, and showing longer filaments in comparison to mocked control cells. Moreover, crizotinib-treated cells appeared multinucleated with evenly stained nuclear fragments (Fig. 2d).

To test the possible effect of crizotinib on microtubule dynamics, ARMS cells were treated with crizotinib for 24 h and examined for spindle morphology by immunofluorescence staining of alpha-tubulin (Fig. 2e). Normal bipolar spindle formation was affected by crizotinib treatment leading to multiple asters with unaligned chromosomes (Fig. 2e) and thus being responsible for the formation of cells with an abnormal size and DNA content. Nocodazole, a prototypic microtubule inhibitor, was used as a positive control for microtubule depolymerisation (Fig. 2e).

**Fig. 3:**
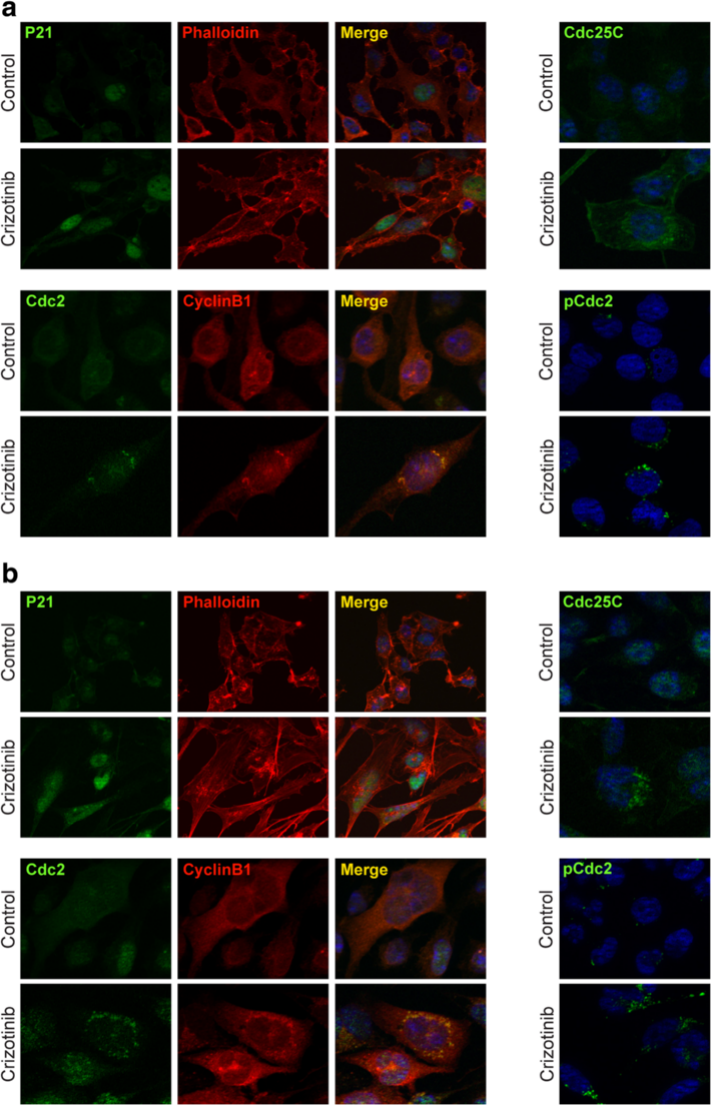
Changes in molecular regulators of cell cycle in RH4 and RH30 cell lines treated with crizotinib. **a** Immunofluorescence experiments showing the expression and localization of p21, Cdc25C, Cdc2, Cyclin B1 and p-Cdc2 (Thr14/Tyr15) proteins in RH4 cells treated or not with 1.5 μM crizotinib for 24 h. **b** Immunofluorescence experiments showing the expression and localization of p21, Cdc25C, Cdc2, Cyclin B1 and p-Cdc2 (Thr14/Tyr15) proteins in RH30 cells treated with 1.5 μM crizotinib for 24 h. Control cells were treated with DMSO. TRITC-phalloidin was used to mark F-actin filaments of cellular cytoskeleton. Images captured under ApoTome microscope at 40× magnification

To investigate the mechanism of the cell cycle perturbations, the impact of crizotinib on the expression and activation status of proteins related to the cell cycle checkpoints was investigated. Western blotting experiments showed that the crizotinib-related G2/M cell cycle arrest was associated with an altered expression of Cyclin B1, Cdc25C, p27 and Cdc2 phosphorylation at Thr14/Tyr15 (the inactive form of Cdc2) in both RH4 and RH30 cells (Fig. 2f). Furthermore, crizotinib exposure affected the expression of the G1/S cell cycle regulators Cdc25A, CDK4 and Cyclin D3 (Fig. 2f). Immunofluorescence experiments confirmed that 1.5 μM crizotinib for 24 h caused over-expression and nuclear staining of p21, a key regulator of cell cycle progression, and a cytoplasmatic retention of Cdc25C, a protein involved in the mitosis entry, in both RH4 and RH30 treated-cells (Fig. 3a-b). Furthermore, ARMS cells exposed to crizotinib showed that Cdc2 and Cyclin B1,two G2/M-regulating proteins, co-localized outside the nuclear portion with a massive staining evident in the perinuclear area (Fig. 3a-b). This finding suggested that the complex Cdc2/Cyclin B1 was not able to enter into the nucleus, this leading to the G2/M arrest assessed by FACS analysis. The marked up-regulation of p-Cdc2 (Thr14/Tyr15) in both cell lines treated with crizotinib confirmed that the Cdc2 protein was in its inactive form (Fig. 3a-b). To our knowledge, this is the first experimental evidence that links crizotinib with G2/M cell cycle arrest and microtubules subcellular organization in ARMS cells.

### Crizotinib induces apoptosis in ARMS cell lines

**Fig. 4:**
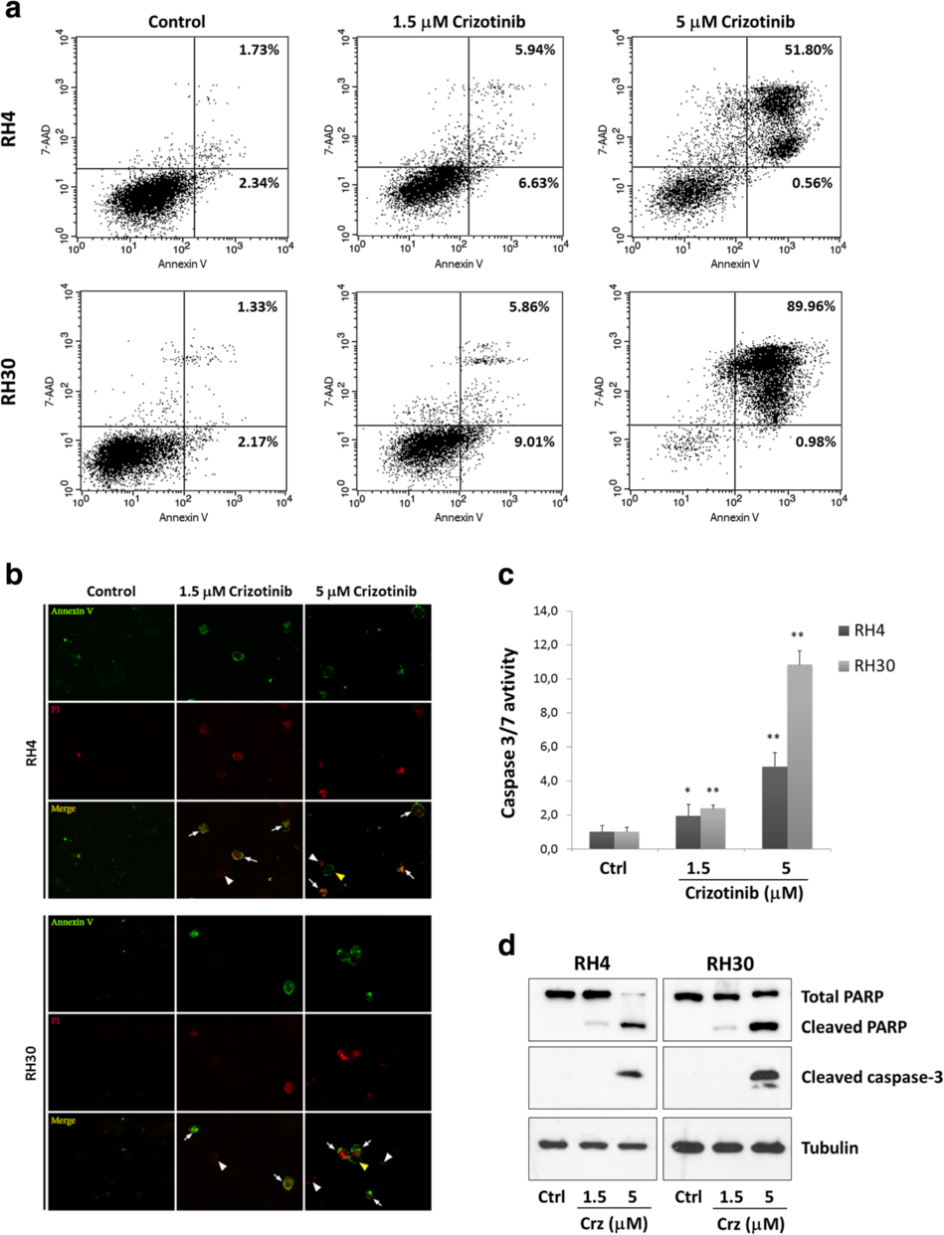
Effects of crizotinib treatment on apoptosis in RH4 and RH30 cell lines. **a** Dot blot graphs show the rate of apoptosis in control and crizotinib-treated cells (1.5 μM and 5 μM) for 48 h. Control cells were treated with DMSO at the maximum amount used to deliver crizotinib. Cells were stained with Annexin V and 7-AAD followed by FACS analysis. The percentage of early and late apoptotic cells is indicated. **b** Apoptotic/necrotic cells in RH4 and RH30 increased in concomitance of increasing doses of crizotinib (1.5 and 5 μM), as demonstrated by Annexin V and PI staining. Cells were observed and photographed under ApoTome fluorescence microscope. Control cells were treated with DMSO. **c** Caspase 3/7 activity is induced in a dose-dependent manner in cells treated with crizotinib for 48 h. Control cells (Ctrl) were treated with DMSO. Histograms represent the mean value ± SD of three independent experiments, each performed in triplicate (*, p<0.05; **, p<0.01). **d** Cleavage of PARP and caspase-3 was evaluated by Western blotting experiments in RH4 and RH30 cells, treated with crizotinib (Crz) as indicated. Control cells (Ctrl) received DMSO vehicle

The induction of apoptosis was investigated as another possible mechanism by which crizotinib inhibits ARMS cell growth. Treatment of RH4 and RH30 cell lines with 1.5 μM crizotinib for 48 h resulted in a moderate increase in the percentage of cells undergoing early (Annexin V positive; low right of the quadrants) and late apoptosis/necrosis (Annexin V/7-AAD positive; upper right of the quadrants) when compared to mock-treated control cells, as assessed by flow cytometry (Fig. 4a). Exposure to 5 μM crizotinib for the same time significantly increased the number of RH4 and RH30 cells in the late stages of apoptosis/necrosis (Fig. 4a). Annexin V-FITC/PI dual staining confirmed that higher drug concentrations caused drastic cytotoxic effects in both ARMS cell lines (Fig. 4b). To address whether the apoptotic process was associated with caspases, the effect of crizotinib on caspase-3/7 proteolytic activity was studied. Luminescence reading using the Caspase-glo 3/7 assay showed that low doses of crizotinib led to moderate levels of caspase 3/7 activation, whilst a more robust activity was observed in response to higher drug concentrations (Fig. 4c). Western blotting experiments confirmed that cleaved PARP expression, the active form involved in the apoptotic process, increased in concomitance with crizotinib concentration (1.5 μM and 5 μM) in both ARMS tumour cell lines, whilst cleaved caspase-3 levels were evident only in 5 μM crizotinib-treated cells (Fig. 4d). Taken together, the present data suggest that crizotinib is able to induce apoptosis in RH4 and RH30 cells via activation of the caspase cascade.

### Crizotinib induces autophagy in ARMS cell lines by increasing ROS accumulation

**Fig. 5:**
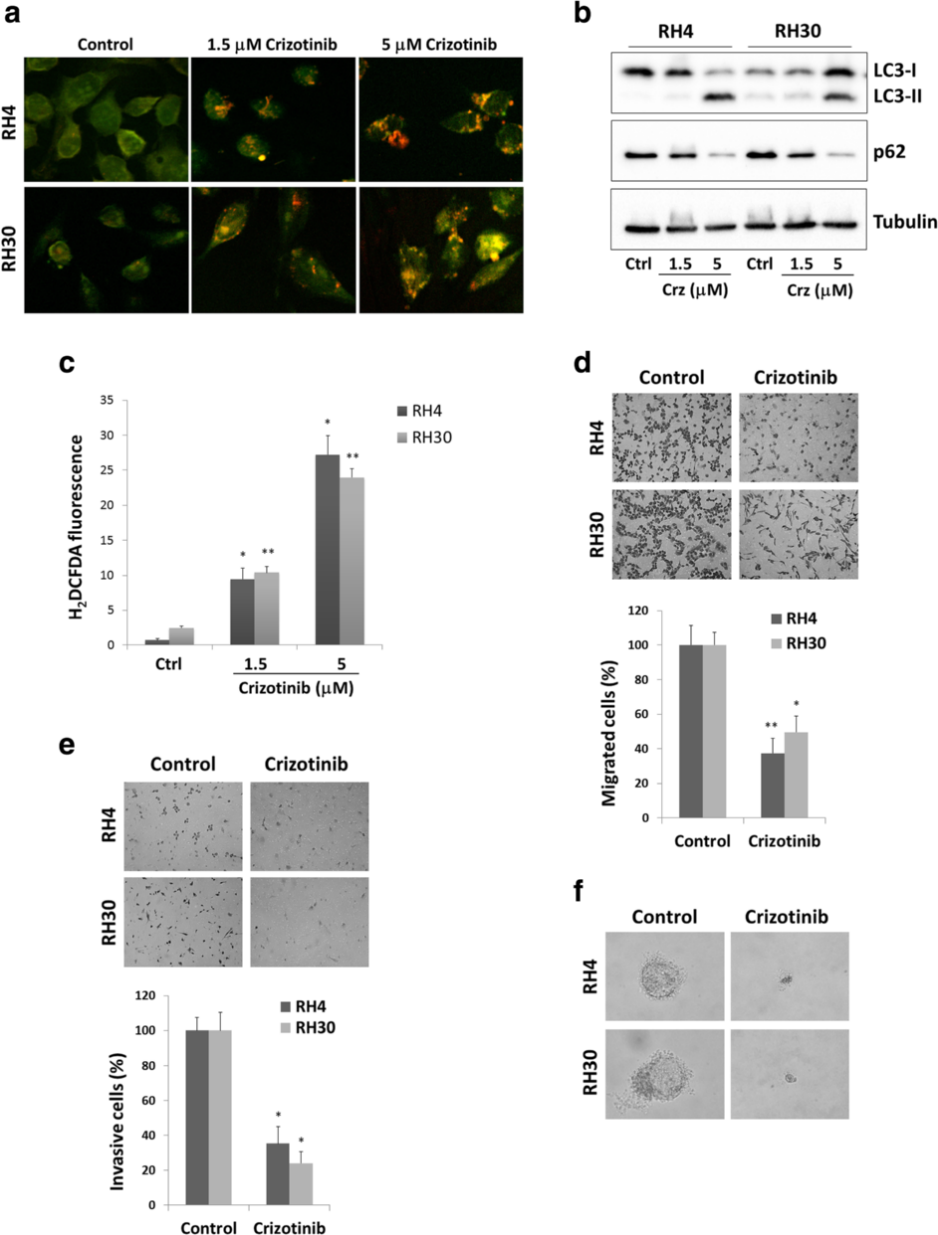
Effects of crizotinib treatment on autophagy, migration, invasion and colony formation in RH4 and RH30 cell lines. **a** Acridine orange staining showed that RH4 and RH30 DMSO-treated control cells exhibited no autophagosomes, whereas crizotinib-treated cells showed abundant cytoplasmic acidic vesicular organelles (AVO) formation, a characteristic of autophagy. Samples were observed under an ApoTome fluorescence microscope. **b** LC3 and p62 protein levels were examined in RH4 and RH30 cells treated with crizotinib (Crz) at various concentrations for 24 h. Control cells (Ctrl) received DMSO vehicle. **c** ARMS cells were treated with crizotinib (1.5 and 5 μM) for 24 h and H_2_DCFDA was added 45 min before collecting cells. H_2_DCFDA fluorescent intensities were analysed by FACSCalibur. Control cells (Ctrl) with H_2_DCDA staining were used as a negative control. Bar charts represent the mean fluorescence ± SD for each sample (*, *p*-value <0.05 compared with the respective negative control; **, *p* < 0.01). Three independent experiments were performed. **d** Crizotinib (1.5 μM) treatment led to a decreased cell migration in both RH4 and RH30 cells. Representative images of migrated cells using the transwell migration assay (crystal violet staining, magnification of 20×). Histograms represent the average values ± SD as determined by counting 8 fields of cells under the microscope. Three independent experiments were performed, each in triplicate (*, *p*-value <0.05 compared with the respective negative control; **, *p* < 0.01). Control cells were treated with DMSO. **e** Crizotinib (1.5 μM) treatment led to decreased cell invasion in both RH4 and RH30 cells. Control cells were treated with DMSO alone. Representative images of invasive cells using the Matrigel-invasion assay (crystal violet staining, 20× magnification). Histograms represent the mean value ± SD as determined by counting 8 fields of cells under the microscope. Three independent experiments were performed, each in triplicate (*, *p* < 0.05). **f** Crizotinib (1.5 μM) induction led to a decreased cell colony formation capacity in both RH4 and RH30 cells. Control cells were treated with DMSO alone. Representative images of a single cell derived-sphere in mocked control and treated cells cultured in soft agar for 21 days (original 40× magnification)

In order to evaluate whether crizotinib induced autophagy in ARMS cells, the formation of autophagosomes was investigated by staining with acridine orange (AO). As shown in Fig. 5a, crizotinib treatment for 24 h induced the accumulation of acidic vescicular organelles (AVOs), which are linked to increased autophagy, in a concentration-dependent manner in both RH4 and RH30 cells. Crizotinib increased levels of LC3-II, a protein widely used for monitoring autophagy, with a concomitant down-regulation of p62/sequestosome 1, a protein involved in the formation and autophagic degradation of polyubiquitin-containing bodies (Fig. 5b). Taken together, these results demonstrate that elevated doses of crizotinib are able to induce autophagy in ARMS cells. Since autophagy is mediated by reactive oxygen species (ROS) accumulation, the ability of crizotinib to increase oxidative potential was monitored using H_2_DCFDA fluorescent dye. ROS levels were evaluated after crizotinib exposure in a concentration-dependent manner in both RH4 and RH30 cell lines (Fig. 5c). Pre-treatment for 1 h with NAC, a thiol antioxidant and ROS scavenger, resulted in a significant reduction of ROS levels after crizotinib addiction (data not shown), further demonstrating the nature of the effect.

### Crizotinib reduces cell migration and invasion ability as well as anchorage-dependent and -independent growth in ARMS cell lines

The assessment of the possible alteration of the migration capacity due to crizotinib in RH4 and RH30 was carried out using a Boyden chamber assay. After 24 h, a smaller number of cells were observed on the bottom side of the membranes in crizotinib-treated cells when compared with controls, indicating that the drug reduced the ability of ARMS cells to migrate across the pores of the Boyden membrane (Fig. 5d). Similarly, crizotinib-treated ARMS cells were less able to invade Matrigel (Fig. 5e). The effects of crizotinib on attachment-independent clonal cell growth in a soft agar assay, a hallmark of cell transformation were also analysed. ARMS untreated cells were able to form colonies in an anchorage-independent manner, while crizotinib drastically reduced this ability, with both cell lines forming smaller colonies (Fig. 5f). The capacity of crizotinib to regulate cell growth was also tested on standard non-pyrogenic polystyrene tissue culture plates for anchorage-dependent (adherent) growth conditions. Highly malignant untreated cells, plated at low concentration, were able to survive, form colonies and proliferate, while crizotinib-treated cells died 5 days after treatment (data not shown).

### Crizotinib inhibits AKT and ERK phosphorylation in ARMS cell lines

**Fig. 6:**
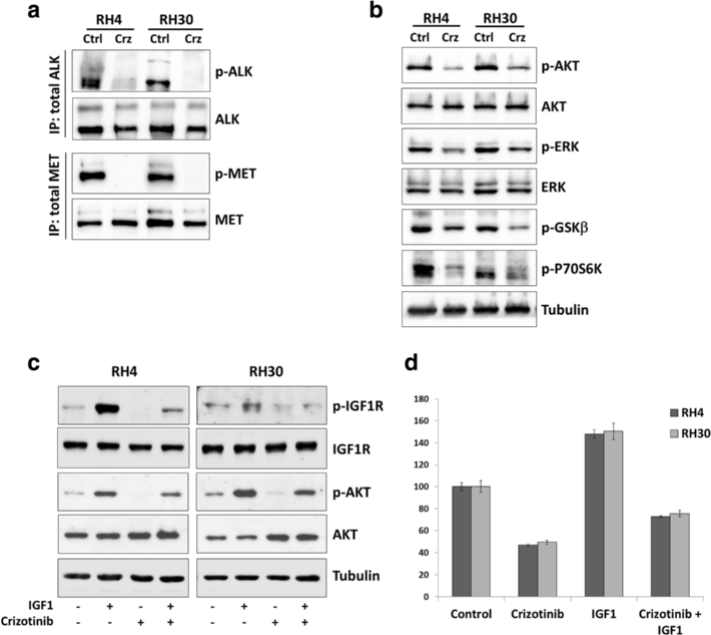
Effects of crizotinib on ALK/MET phosphorylation, AKT and ERK activation and cell growth through IGF-1 signalling in RH4 and RH30 cell lines. **a** Immunoprecipitation and western blot showing expression of p-ALK, total ALK and p-MET and total MET in RH4 and RH30 cells treated with or without 1.5 μM crizotinib (Crz) for 24 h. Control cells (Ctrl) were treated with DMSO alone. **b** Western blotting analysis of phosphorylation levels of AKT and ERK proteins and downstream molecules GSKβ and P70S6K in RH4 and RH30 cells treated with or without 1.5 μM crizotinib (Crz) for 24 h. Control cells (Ctrl) were treated with DMSO alone. **c** Western blotting analysis of phosphorylation levels of IGF1R and AKT proteins in IGF1-treated cells with or without crizotinib. RH4 and RH30 cells were starved in serum deficient medium for 24 h prior to induce with IGF1 (50 ng/ml) for 30 min. Crizotinib (1.5 μM) was added 4 h before ligand stimulation. **d** MTT proliferation assay in ARMS cells grown in the presence or absence of IGF1 (50 ng/ml) and crizotinib (1.5 μM) for 48 h. Bars represent the mean values for nine replicate wells ± SD and are representative of three independent experiments

To gain insight into the molecular mechanisms underlying crizotinib effects on ARMS cells, ALK and MET activation in treated RH4 and RH30 cells were firstly evaluated. Phosphorylated levels of both ALK and MET proteins were markedly reduced by crizotinib addition in both cell lines, as revealed by immunoprecipitation experiments with the appropriate antibody and the following hybridization with p-ALK (Tyr1604) and p-MET (Tyr1234/Tyr1235), respectively (Fig. 6a). Similarly, the expression of phospho-AKT (p-AKT) and phospho-ERK (p-ERK), two important signalling molecules involved in cancer cell growth and survival, was also evaluated in RH4 and RH30 treated with 1.5 μM crizotinib for 24 h. Crizotinib exposure led to a robust dephosphorylation of AKT without affecting its total levels (Fig. 6b), whilst p-ERK levels showed a less marked reduction (Fig. 6b). Furthermore, the phosphorylation level of GSKβ and P70S6K downstream molecules was substantially reduced by crizotinb treatment (Fig. 6b), providing evidence that this compound affects aberrant cell signalling in ARMS cells by blocking AKT- and ERK-related pathways.

To investigate the effect of suppressing ALK or MET expression in RH4 and RH30 cells, we used specific RNA duplexes directed against the ALK or MET coding regions. A sequence against the *C. elegans* (NC siRNA) was used as a negative control. Western blotting analysis revealed that ALK protein was reduced in ALK siRNA transfected cells but not in NC siRNA samples (Additional file 1A). Similarly, MET specific knock-down was obtained only in MET siRNA-transfecting cells (Additional file 1B). Phosphorylated and total levels of AKT and ERK kinases were not significantly modified by ALK or MET siRNA at 48 h after transfection (Additional file 1A and B). MET siRNA delivery was able to induce a modest G2/M arrest in both RH4 (G2/M: 21.5 % MET siRNA *vs.* 13.3 % NC siRNA) and RH30 (G2/M: 13.2 % MET siRNA *vs.* 9.1 % NC siRNA) transfected cells, whilst ALK siRNA alone did not significantly affect the percentage of cells in the G2/M phase (12.4 % in ALK siRNA *vs.* 13.3 % in NC siRNA RH4 cells; 8.0 % in ALK siRNA *vs*. 9.1 % in NC siRNA RH30 cells). When ARMS cells were transfected for 48 h with ALK, MET or NC siRNA, apoptosis was respectively: 15.6, 14.1 and 12.0 % for RH4, and 14.8, 13.9 and 10.2 % for RH30 cancer cells, respectively, showing that single targeting of ALK or MET proteins did not produce notable programmed cell death. These data suggest that ALK and MET are not the sole targets of crizotinib altering ARMS cellular phenotype.

### Crizotinib inhibits both IGF1-induced signalling and cell growth in ARMS cell lines

Since IGF1R, a tyrosine kinase receptor constitutively activated and highly expressed in ARMS cells, share structural homologies [34] with ALK and MET proteins, the possible role of crizotinib on IGF1R phosphorylation was explored. Indeed, drug treatment markedly inhibited p-IGF1R levels and the IGF1-mediated signal transduction pathway (Fig. 6c). After ligand stimulation by IGF1, a strong increase of phosphorylated IGF1R (p-IGF1R) levels was observed in both RH4 and RH30. Crizotinib pre-treatment for 4 h efficiently inhibited the IGF1-mediated stimulation of p-IGFIR with a parallel decrease of the known downstream effector p-AKT in both ARMS cell lines, without the respective total protein levels being perturbed (Fig. 6c). Crizotinib was also able to block the IGF1-induced cell growth of RH4 and RH30 as shown by MTT cell proliferation assays (Fig. 6d). Cells were starved and then allowed to grow for 48 h in the presence of IGF1, with or without crizotinib. Crizotinib-treated cells showed a decreased cell proliferation in starved conditions and in the presence of IGF1 compared with crizotinib-untreated cells (Fig. 6d). The present findings show for the first time that crizotinib may also act on IGF1R activity, thus circumventing possible resistance mechanisms driven by IGF1R signalling observed in RMS tumours.

## Discussion

Recent clinical studies have reported a promising therapeutic impact of crizotinib, an ATP-competitive inhibitor of ALK/MET activity, in the treatment of ALK- and/or MET-positive tumours [25, 35]. However, its antitumor properties in RMS, the most common soft tissue sarcoma in children [1, 2], have only recently been questioned [11]. The growing interest in the expression and oncogenic activity of ALK in RMS [8, 10, 11] implies a possible therapeutic role of crizotinib in this malignancy. Accordingly, the present study investigated the possible effects and molecular mechanisms of crizonitib on RH4 and RH30 human ARMS cell lines, which over-express ALK and MET genes at mRNA and protein levels (Fig. 1). The crizotinib-treated cells displayed a significant decrease in proliferation and viability at clinically relevant concentrations (≤5 μM) [25]. Interestingly, the reduced cell growth in both tumour cell lines was primarily associated with cell cycle arrest and apoptosis/autophagy, in accordance with a very recently published article by Peron et al. [11]. This study highlighted that after crizotinib exposure, RH4 and RH30 cells accumulated in G2/M phase and showed morphological alterations, such as enlarged or multiple nuclei, characteristic of defective cell division (Fig. 2c-d), which was attributable to the deregulation of important cell cycle regulators. In particular, crizotinib treatment led to the down-regulation of Cyclin D3 expression and to the over-expression of p21/p27 proteins. To this regard, Cyclin D3 activity associated with CDK4 has been reported to be necessary for cell cycle progression through G2 phase into mitosis after UV radiation [36]. More specifically, crizotinib caused cytoplasmatic retention of Cdc25C protein and concomitant hyper-phosphorylation of Cdc2 at Thr14/Tyr15, while Cyclin B1 became overexpressed and predominantly accumulated around the nuclear envelope. Taken together, these data suggest that crizotinib activates the G2/M checkpoint in ARMS cells by sequestering Cdc25C in the cytoplasm and thus promoting the phosphorylation of Cdc2, which is not able to form an active complex with Cyclin B1, this preventing the entrance in the nucleus and so stalling the mitosis as reported in other cancers [37, 38]. Furthermore, cell cycle perturbation might also be related to the crizotinib ability of altering cellular microtubule dynamics, as revealed by the formation of multipolar spindles (Fig. 2e).

The biological mechanisms altered by crizotinib also include the modulation of signal transduction molecules linked to the ALK/MET system, such as AKT and ERK, which are involved in cell proliferation, adhesion, motility, differentiation and survival [39–41]. In both ARMS cell lines, crizotinib (at 1.5 μM) reduced ALK and MET activation and caused a significant reduction in the phosphorylation levels of AKT (Ser473), even if it was less effective in suppressing ERK activation. The marked inhibitory effect on p-AKT expression might explain the profound alterations in cell proliferation and migration/invasion observed in these experiments, as suggested by the critical role of the PI3K/AKT/mTOR cascade in DNA synthesis, G2/M transition [42–44] and invasion of RMS cells [45]. The minor effect on ERK phosphorylation would explain why crizotinib is less effective in inducing caspase-dependent apoptosis in RH4 and RH30 cells. These findings are consistent with previous studies [46, 47] showing that a massive and concomitant block of both PI3K/AKT/mTOR and RAS/MEK/ERK cascades induces significant cytotoxicity in RMS cell lines, while the individual inhibition of each pathway is not sufficient to trigger apoptosis. Indeed, 1.5 μM concentration of crizotinib moderately increased programmed cell death, which dramatically enhanced only at higher concentrations (i.e., 5 μM) as demonstrated by the typical apoptotic morphology, increased early/late apoptotic cell population and activation of PARP and caspase-3 in both RH4 and RH30 (Fig. 4). Notwithstanding, high dose of crizotinib led to an increased autophagic flux, as demonstrated by autophagosome formation, augmented accumulation of LC3-II and diminished levels of p62, this proving the existence of an alternative death mechanism by which this compound exerts its antitumor effects on RMS cells. Indeed, the role of autophagy in accelerating cell death seems to be context-dependent and recent literature reports situations where autophagy is related to cytotoxic effects and senescence in cancer cells [48, 49]. Furthermore, as for many other anticancer agents [50, 51], the excessive generation of ROS seems to be the mechanism involved in the cytotoxic activity of crizotinib, with endogenous ROS levels proportionally increasing with the drug concentration (Fig. 5c). Thus, crizotinib appears to have multiple outcomes depending on the tumour cell type or treatment conditions, such as dose-concentrations. Surprisingly, ALK and MET silencing by RNAi did not produce notable cell-cycle arrest or apoptosis, indicating that crizotinib exposure is more effective in blocking ALK and MET signalling than single siRNA transfection. Even if the modest effect on cell cycle and survival in RH4 and RH30 cells might be also due to fact that we performed siRNA transient transfection, new evidences suggest that crizotinib may have unidentified off-target antitumor effects which, independently of ALK and/or MET, might contribute to the cellular and molecular alterations observed in ARMS cells [11, 52]. Indeed, non-MET related effects of crizotinib were reported to contribute to the cell-cycle arrest and cytotoxicity observed in thyroid cancer cells [52] and inhibition of other RTKs, independently of ALK expression and activity, was recently observed in RMS cells [11]. To this concern, the present study found that crizotinib was able to decrease phosphorylation levels of intracellular IGF1R and cell proliferation in response to IGF1 ligand stimulation (Fig. 6c), this suggesting that the drug exerts negative effects on IGF1R signalling system. The IGF1/IGF1R axis is overexpressed and constitutively phosphorylated especially in PAX3/7-FOXO1 positive ARMS cells, this being one of the major tumorigenic pathways in this tumour [53–55]. Whether crizotinib inhibits IGF1R directly by binding to the cell surface receptor or indirectly by a possible functional interaction between ALK and IGF1R is an area of current research. Indeed, common activation and regulatory mechanisms are believed to exist for members of the RTK superfamily, comprehending ALK, MET and IGF1R proteins [34], most likely due to their similar nature. Likewise, recent data have shown that IGF1R tyrosine kinase and the NPM-ALK oncogene associate and reciprocally increase their phosphorylation and activation to induce survival of T-cell ALK-positive anaplastic large-cell lymphoma cells [56, 57]. In line with these observations, IGF1R, as well as MET and EGFR, sensitivity to ALK inhibitors has just been demonstrated by Peron et al. [11].

Collectively, our data show that crizotinib-dependent induction of the G2/M checkpoint significantly contributes to the *in vitro* reduction of growth, survival and clonogenicity of RH4 and RH30 cells. Even if crizotinib was initially reported as an ALK and MET inhibitor, this study support the evidence that this drug may exert its effects by also affecting the activity of other RTKs showing structural analogies in some of their domains, such as IGF1R, and so a broader pool of downstream molecular targets [52]. Future studies will be needed to better understand these additional signalling pathways and the interplay among cascade components as well as to monitor the *in vivo* crizotinib effects on RMS growth. The current treatment for RMS is a combination of surgery, chemotherapy and radiotherapy. However, the development of resistance to chemotherapy and radiotherapy is often a significant limiting factor, leading to therapeutic failures and poor survival [58]. As previous studies suggested that the inhibition of ALK and IGF1R pathways may potentiate chemotherapy and radiotherapy in lung cancer [59, 60], crizotinib might be an effective additional therapeutic agent in patients with aggressive ARMS tumours that overexpress ALK, MET and IGF1R proteins.

## Conclusions

The exposure to crizotinib causes the individual or synergistic ALK, MET and IGF1R inhibition, and thereafter results in the down-regulation of AKT signal pathway, which eventually leads to the cell cycle arrest, inhibition of migration/invasion and induction of apoptosis observed in ARMS cell lines.

## Additional file

Additional file 1:
**ALK and MET knock-down by RNA interference.** A: RH4 and RH30 cells were transfected with either scramble control siRNA (NC siRNA) or ALK siRNA. Cells were harvested 48 h after transfection and ALK, AKT (phosphorylated and total protein) and ERK (phosphorylated and total protein) levels were analysed by Western blotting. B: RH4 and RH30 cells were transfected with either scramble negative control siRNA (NC siRNA) or MET siRNA. Cells were harvested 48 h after transfection and MET, AKT (phosphorylated and total protein) and ERK (phosphorylated and total protein) levels were analysed by Western blotting. Tubulin was used as loading control in all experiments. (TIFF 258 kb)
