# Supplementary figures and images for: Crizotinib-induced antitumour activity in human alveolar rhabdomyosarcoma cells is not solely dependent on ALK and MET inhibition
Source: J Exp Clin Cancer Res. 2015 Oct 6;34:112. doi: 10.1186/s13046-015-0228-4 (PMC4596370; doi:10.1186/s13046-015-0228-4)

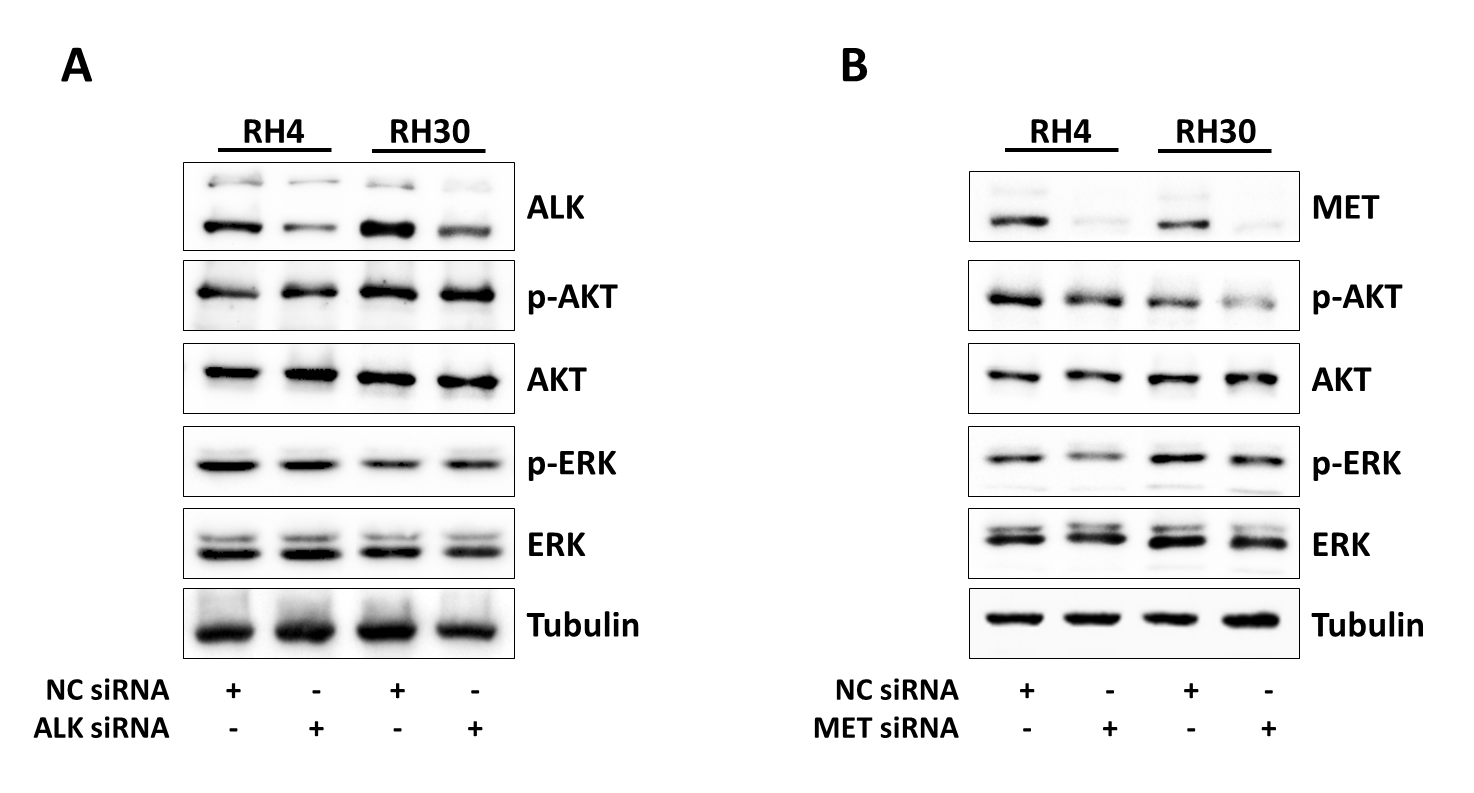

Supplement: Additional file 1: — ALK and MET knock-down by RNA interference. A: RH4 and RH30 cells were transfected with either scramble control siRNA (NC siRNA) or ALK siRNA. Cells were harvested 48 h after transfection and ALK, AKT (phosphorylated and total protein) and ERK (phosphorylated and total protein) levels were analysed by Western blotting. B: RH4 and RH30 cells were transfected with either scramble negative control siRNA (NC siRNA) or MET siRNA. Cells were harvested 48 h after transfection and MET, AKT (phosphorylated and total protein) and ERK (phosphorylated and total protein) levels were analysed by Western blotting. Tubulin was used as loading control in all experiments. (TIFF 258 kb) [file 13046_2015_228_MOESM1_ESM.tif]
